# Prognostic value of CtIP/RBBP8 expression in breast cancer

**DOI:** 10.1002/cam4.141

**Published:** 2013-10-03

**Authors:** Isabel Soria-Bretones, Carmen Sáez, Manuel Ruíz-Borrego, Miguel A Japón, Pablo Huertas

**Affiliations:** 1 Centro Andaluz de Biología Molecular y Medicina Regenerativa (CABIMER)Av. Americo Vespucio s/n, Sevilla, 41092, Spain; 2 Instituto de Biomedicina de Sevilla (IBIS), Hospital Universitario Virgen del Rocío/CSIC/Universidad de SevillaAvenida Manuel Siurot s/n, Sevilla, 41013, Spain; 3 Oncology Unit, Hospital Universitario Virgen del RocíoAvenida Manuel Siurot s/n, Sevilla, 41013, Spain; 4 Department of Pathology, Hospital Universitario Virgen del RocíoAvenida Manuel Siurot s/n, Sevilla, 41013, Spain; 5 Department of Genetics, University of SevillaAv. Reina Mercedes s/n, Sevilla, 41080, Spain

**Keywords:** Breast cancer, CtIP/RBBP8, DNA repair, retinoblastoma

## Abstract

CtIP/RBBP8 is a multifunctional protein involved in transcription, DNA replication, DNA repair by homologous recombination and the G1 and G2 checkpoints. Its multiple roles are controlled by its interaction with several specific factors, including the tumor suppressor proteins BRCA1 and retinoblastoma. Both its functions and interactors point to a putative oncogenic potential of CtIP/RBBP8 loss. However, CtIP/RBBP8 relevance in breast tumor appearance, development, and prognosis has yet to be established. We performed a retrospective analysis of CtIP/RBBP8 and RB1 levels by immunohistochemistry using 384 paraffin-embedded breast cancer biopsies obtained during tumor removal surgery. We have observed that low or no expression of CtIP/RBBP8 correlates with high-grade breast cancer and with nodal metastasis. Reduction on CtIP/RBBP8 is most common in hormone receptor (HR)-negative, HER2-positive, and basal-like tumors. We observed lower levels of RB1 on those tumors with reduced CtIP/RBBP8 levels. On luminal tumors, decreased but not absence of CtIP/RBBP8 levels correlate with increased disease-free survival when treated with a combination of hormone, radio, and chemo therapies.

## Introduction

Cancer appearance, development, and progression are characterized by a progressive accumulation of genetic mutations that abolish the natural constrains of cellular division in pluricellular organisms [1, 2]. While in normal cells mutations build up slowly due to the existence of multiple mechanisms that prevent them, DNA mutations at accelerated rates in precancerous and cancer cells accumulate, a phenomenon known as genomic instability [1, 2]. There are many reasons why the genome of cancer cells is less stable. First, they tend to replicate more and faster than normal cells, mainly due to the loss of cell cycle proteins, such retinoblastoma (RB1), that control the G1/S transition [1, 3, 4]. Second, replication tends to be more mutagenic in those cells, either due to problems in the replication machinery itself or in the response to replication-borne DNA lesions [2, 5]. Third, cancer and precancer cells usually deal erroneously with damaged DNA, either not signaling the lesions correctly (e.g., due to the lack of p53, RB1, ataxia telangiectasia mutated (ATM), ataxia telangiectasia and Rad3 related (ATR), etc.), or due to alterations on the DNA repair mechanisms (caused by mutations such as BRCA1, BRCA2, and RAD51) [6, 7]. Finally, a more tenuous source of genomic instability is the general upheaval of transcription profiles in cancer cells, which contribute to increased mutations [8]. Considering all, it is not surprising that mutations that affect any of the aforementioned mechanisms of replication, repair, and transcription are usually selected early on in cancer development and have a net contribution of cancer progression.

A protein with a strong but not completely explored cancer connection is CtIP/RBBP8. This protein has been implicated in several nuclear pathways, mainly through its interaction with additional factors, many of them bona fide tumor suppressor genes. CtIP/RBBP8 was first described as a transcriptional corepressor together with CtBP [9]. It has also been shown to bind several other cancer-related transcription factors such as Ikaros, TRB3, or LMO4 [10–12]. At the same time, CtIP/RBBP8 was described to play a critical role in the initiation of S-phase and DNA replication as a negative regulator of RB1 and the G1 checkpoint [13, 14]. Later, CtIP/RBBP8 was found to participate in DNA replication by binding proliferating nuclear cell antigen (PCNA) and to play an important role in minimizing replication-induced DNA breaks [15]. Finally, CtIP/RBBP8 plays a critical role in DNA damage detection, signaling, and repair, mainly by its interactions with BRCA1 and the MRE11-RAD50-NBS1 (MRN) complex [16–18].

Despite these connections with cancer, little is known about the role of CtIP/RBBP8 as a tumor suppressor gene itself. CtIP/RBBP8 microsatellite-induced frameshift mutations have been found in colorectal cancer [19, 20] and endometrial cancer [21], and point mutations have been observed in some cancer cell lines [18]. Strikingly, haploid insufficiency in mouse led to increased tumor appearances, of mainly large B cell lymphomas [13]. Functionally, CtIP/RBBP8 transcriptional activity seems to contribute to cancer development and treatment success [22–24]. In addition, CtIP/RBBP8 is a key player in cell cycle control, through its interaction with RB1, as its activity is required for overcoming RB1-mediated cell cycle arrest [13, 14]. The relationship between both factors is so tight that CtIP/RBBP8 knock-out in mouse is lethal in the presence of functional RB1 [13].

The stronger link between CtIP/RBBP8 and cancer, and specifically breast cancer, relies in its functional interaction with BRCA1 in DNA repair. CtIP/RBBP8 and BRCA1 physically interact and act together in the homologous recombination pathway [17, 18, 25, 26]. In fact, BRCA1 point mutations that abolish its interaction with CtIP/RBBP8 have been associated with tumor progression [27–29]. In stark contrast, other mutations in conserved BRCA1 regions that maintain CtIP/RBBP8 binding are considered benign [30]. Despite this relationship, no CtIP/RBBP8 mutations have been observed so far in families with hereditary cancer but that are wild type for BRCA1 and BRCA2 [31]. However, an association with specific CtIP/RBBP8 haplotypes has been proposed to be a cancer-risk modifier in breast cancer for BRCA1 mutation carriers [32], but not for ovarian cancer [32, 33].

Despite all the available data, no systematic study of CtIP/RBBP8 expression on breast cancer samples and its correlation with treatment response and disease-free survival has been done. We have now performed this study using 384 paraffin-embedded breast cancer biopsies obtained during tumor removal surgery between 2004 and 2007 at a single institution. We have analyzed CtIP/RBBP8 and RB1 presence on all tumor samples and correlated their expression levels with cancer prognosis markers. We observed a significant link between CtIP/RBBP8 and RB1 expression, as well as a strong relationship between CtIP/RBBP8 levels and specific breast cancer types. From this, we conclude that patients with luminal cancer who have decreased CtIP/RBBP8 expression respond better to the combined hormone therapy, radiotherapy, and chemotherapy treatment than patients with normal or no CtIP/RBBP8 expression.

## Patients and Methods

### Patients and tissue sampling

A total of 384 formalin-fixed paraffin-embedded (FFPE) samples of invasive breast carcinomas were obtained from patients diagnosed from July 2004 to July 2007 at University Hospital Virgen del Rocío, Sevilla, Spain. The Ethical Committee of the Hospital approved the study. No consent from the patients was needed.

All biopsies were stained with anti-CtIP/RBBP8 and anti-RB1 antibodies, but not all samples could be included for every correlations made in this study due to the absence of data related to tumoral classification or disease-free survival after the treatment.

Clinicopathologic data, including age, local and distant metastasis, regional lymph node metastasis, histologic grade, tumor size, tumoral markers, treatment and survival, were obtained from medical records (Table 1).

**Table 1:** Patient description

	*n*=384	%
Tumor		
T1	136	35.4
T2	160	41.7
T3	32	8.3
T4	11	2.9
n.d.	45	11.7
Node		
Negative	165	43.0
Positive	157	40.9
Unknown	62	16.1
Grade		
1	41	10.7
2	140	36.5
3	178	46.4
n.d.	25	6.5
ER		
Negative	91	23.7
Positive	267	69.5
n.d.	26	6.8
PR		
Negative	135	35.2
Positive	223	58.1
n.d.	26	6.8
HER2		
Negative	300	78.1
Positive	65	16.9
n.d.	19	4.9
Ki67		
Negative	77	20.1
Positive	243	63.3
n.d.	64	16.7
Phenotype		
Luminal A	70	18.2
Luminal B HER2−	145	37.8
Luminal B HER2+	23	6
HER2+ (nonluminal)	33	8.6
Triple negative	52	13.5
Unknown	61	15.9
Treatment		
Adjuvant	(300)	78.1
Chemotherapy	183	
Hormonal	230	
Radiotherapy	202	
Anti-HER2	32	
Neoadjuvant	(17)	4.4
Chemotherapy	15	
Hormonal	2	
Radiotherapy	1	
Anti-HER2	1	
No treatment	13	3.4
Unknown	54	14.1
Gender		
Female	381	99.2
Male	3	0.8

ER, estrogen receptor; PR, progesterone receptor; HER2, human epidermal growth factor receptor-2.

Three of the patients included in this study were men; the male tissue samples were considered in the same way as the female samples. The median age was 63 years (range, 27–91 years). The mean follow-up period was 67 months (median, 78 months; range, 1–106 months). Nine samples corresponded to relapsed breast tumors, and these cases were not included in the disease-free survival study. Similarly, patients were treated with chemotherapy before surgery was excluded from the correlations.

### Immunohistochemical staining and scoring

Paraffin-embedded tissue cores (1 mm) were used to build four tissue microarrays, each containing 96 samples that were sectioned at 4 μm. Immunohistochemical staining was carried out to visualize the CtIP/RBBP8- and RB1-positive cells. Paraffin sections were dewaxed in xylene and rehydrated through a graded ethanol series. Antigen retrieval for CtIP/RBBP8 staining was performed with 4N HCl at room temperature for 15 min, followed by 1 mg/mL trypsin at 37°C for 15 min. RB1 samples were heated in Target retrieval solution, pH 9 (Dako, Glostrup, Denmark), using a microwave at 600 W for 20 min. Tissue sections were subsequently immersed in 3% H_2_O_2_ aqueous solution for 30 min to exhaust endogenous peroxidase activity, and then covered with blocking reagent (Roche, Mannheim, Germany) to avoid nonspecific binding. Tissue sections were incubated with primary antibodies overnight at 4°C, using CtIP/RBBP8 mouse monoclonal antibody 1:50 (R. Baer, Columbia University, New York, NY) and RB1 rabbit polyclonal antibody 1:750 (ab39689; Abcam plc, Cambridge, U.K.). Peroxidase-labeled secondary reagents and 3,3′-diaminobenzidine were applied according to the manufacturer's protocols (EnVision™ FLEX for RB1 and EnVision™ FLEX Mouse [Linker] for CtIP/RBBP8, Dako). Slides were then counterstained with hematoxylin and mounted in DPX (BDH Laboratories, Poole, U.K.). Immunostaining was evaluated independently by two observers and scored as follows: CtIP/RBBP8 0, no nuclear staining; 1, moderate; 2, strong; RB1 0, no nuclear staining; 1, intermediate nuclear staining; and 2, strong nuclear staining.

### Statistical analysis

The association between CtIP/RBBP8 expression and clinicopathological features was examined by the chi-square test. Disease-free survival curves were calculated by the Kaplan–Meier method. Survival curves were compared by the log-rank test of Mantel and Haenszel. Calculations were performed using Prism 5.0 (GraphPad, San Diego, CA).

### Breast cancer subtyping according to estrogen receptor (ER), progesterone receptor (PR), and human epidermal growth factor receptor-2 (HER2) status

The breast tumor samples were classified into five subtypes of luminal A (ER+ and/or PR+, HER2− and Ki-67 low), luminal B HER2 negative (ER+ and/or PR+, HER2− and Ki-67 high), luminal B HER2 positive (ER+ and/or PR+, HER2 overexpressed or amplified, and any Ki-67), HER2 positive (nonluminal) (HER2 overexpressed or amplified, ER− and PR−), and triple negative (ER−, PR−, and HER2−), according to the system for the immunohistochemical subtyping of breast cancer [34].

## Results

To analyze a potential CtIP/RBBP8 relationship with breast cancer clinicopathological variables, we analyzed 384 biopsies obtained during tumor removal surgery between 2004 and 2007 at the University Hospital Virgen del Rocío, Sevilla, Spain. Three of the patients were men (Table 1). The age of the patients range from 27 to 91, with a median of 63. All the clinicopathological variants studied are described in Table 1. More than 77% of the samples corresponded to low stage (T1 or T2), and over 70% were hormone receptor (HR) positive. Only 17% were positive for HER2, 62% were luminal cancers, and only 13.5% were triple negative. Grade 3 tumors (49.6%) were more represented than grade 2 (39%) or grade 1 (11.4%). The proliferation marker Ki67 was present in 63.3% of the samples. A summary of the different treatments the patients received is also shown in Table 1.

**Figure 1:**
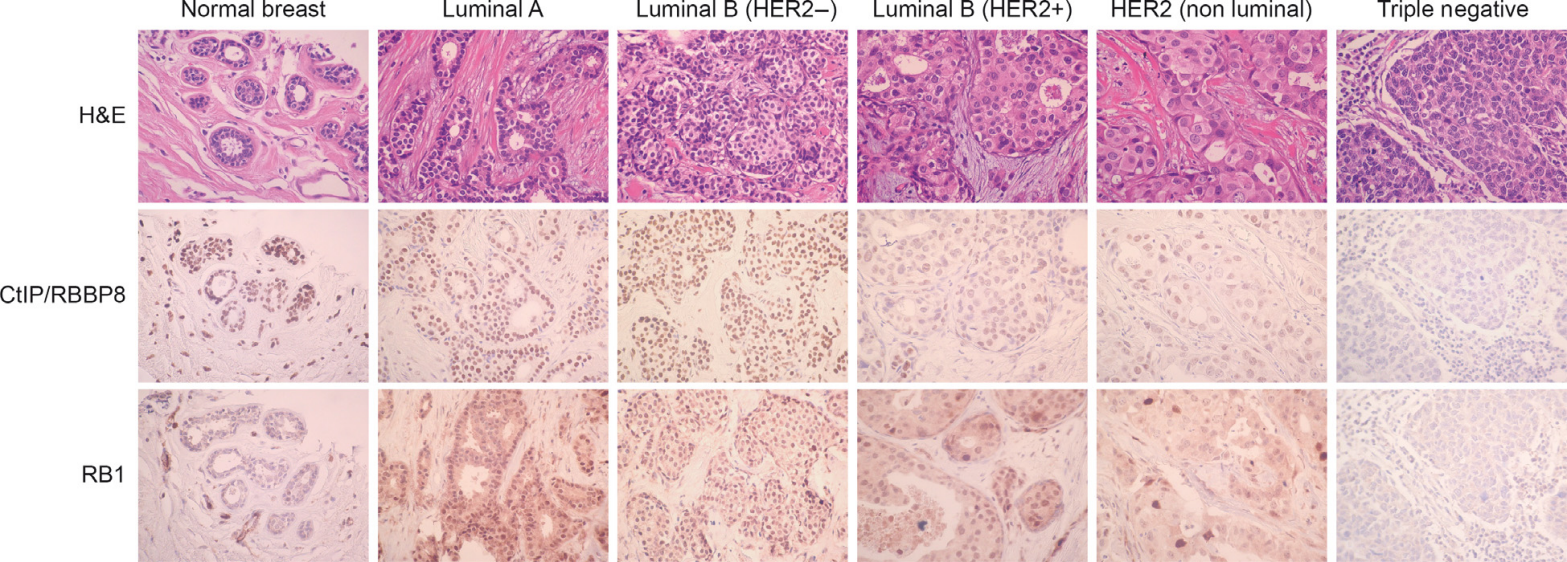
CtIP/RBBP8 and retinoblastoma (RB1) expression in breast cancer biopsies. Paraffin-embedded breast cancer biopsies were immunostained with CtIP/RBBP8 or RB1 antibodies and stained with hematoxylin and eosin as described in the Patients and Methods section. A representative image of each cancer subtype is shown. In addition, the gradation of CtIP/RBBP8 and RB1 protein level can be observed from 2 (normal; left) to 0 (triple negative; right).

We performed an immunohistochemistry study of the CtIP/RBBP8 levels in the paraffin-embedded samples (Fig. 1). We detected three different CtIP/RBBP8 levels (Fig. 1): level 0 contained samples in which no CtIP/RBBP8 could be detected with the antibody; level 1 represented an intermediate level; and level 2 corresponded to samples with a clear nuclear signal of CtIP/RBBP8 in more than 90% of the cells of the tumor. The specificity of the antibody for immunostaining was previously showed [26] and can be observed in Figure S1. Based on mRNA studies, it has been proposed that CtIP/RBBP8 might be overexpressed in certain cancers [12, 35], hence it was important to unequivocally determine which of the three levels corresponded to basal, nonpathological CtIP/RBBP8 protein expression. We thus used six nontumors samples from breast reduction surgery to establish the normal CtIP/RBBP8 expression level on healthy mammary glands (Figs. 1 and S2). For these, we observed that a strong nuclear staining was readily observed in ductal cells in all cases, despite some variations within samples. Based on this, we concluded than normal tissue has an expression level equivalent to level 2. Thus, we considered all samples with a level 2 expression of CtIP/RBBP8 to be normal, and those with level 1 to represent downregulation of the protein. Level 0 corresponded to a complete lack of CtIP/RBBP8. Importantly, we did not find any tumor sample that overexpressed CtIP/RBBP8 in the cohort.

After characterizing all samples for their CtIP/RBBP8 levels, we analyzed the correlation with the clinicopathological variations listed in Table 1. We found that CtIP/RBBP8 expression affects all the given parameters in a statistically significant way (Table 2). Lower levels of CtIP/RBBP8 correlated with more aggressive and advanced clinical characteristics and usually corresponded to grade 3 tumors that had a higher probability to present node metastasis and were less likely to be at the T1 stage. Normal CtIP/RBBP8 level cancers are usually grade 2, at a lower stage (T1), and less likely to present node metastasis. Whereas we found a strong bias for tumors with normal CtIP/RBBP8 levels to be positive for ER and PR receptors (of 91.5% and 78%, respectively), breast cancers with no CtIP/RBBP8 staining had an almost equal distribution of HR-positive and HR-negative samples. In addition, the proportion of HER2+ tumors was significantly higher in tumors expressing low or no CtIP/RBBP8. Moreover, the lower the level of detected CtIP/RBBP8, the higher the proliferation of the cells (as measured by the presence of Ki67).

**Table 2:** CtIP/RBBP8 expression in breast cancer and its relationship with clinicopathological variables

	Absence	Low	Normal	*P*-value
Tumor				
T1	46 (33.3)	51 (45.5)	36 (50.0)	0.032
T2	71 (51.4)	43 (38.4)	34 (47.2)	
T3	16 (11.6)	13 (11.6)	1 (1.4)	
T4	5 (3.6)	5 (4.5)	1 (1.4)	
Node				
Negative	69 (47.9)	52 (47.3)	44 (64.7)	0.044
Positive	75 (52.1)	58 (52.7)	24 (35.3)	
Grade				
1	9 (5.8)	15 (12.2)	17 (20.7)	<0.0001
2	53 (34.4)	42 (34.1)	45 (54.9)	
3	92 (59.7)	66 (53.7)	20 (24.4)	
ER				
Negative	64 (41.8)	20 (16.3)	7 (8.5)	<0.0001
Positive	89 (58.2)	103 (83.7)	75 (91.5)	
PR				
Negative	75 (49.3)	42 (33.9)	18 (22)	0.0001
Positive	77 (50.7)	82 (66.1)	64 (78)	
HER2				
Negative	123 (77.8)	102 (81)	75 (92.6)	0.017
Positive	35 (22.2)	24 (19)	6 (7.4)	
Ki67				
Negative	23 (16.1)	31 (29.0)	23 (32.9)	0.009
Positive	120 (83.9)	76 (71)	47 (67.1)	
Phenotype				
Luminal A	22 (15.7)	27 (23.9)	21 (30)	<0.0001
Luminal B HER2−	53 (37.9)	51 (45.1)	41 (58.6)	
Luminal B HER2+	7 (5)	15 (13.3)	1 (1.4)	
HER2+	22 (15.7)	6 (5.3)	5 (7.1)	
Triple negative	36 (25.7)	14 (12.4)	2 (2.9)	

ER, estrogen receptor; PR, progesterone receptor; HER2, human epidermal growth factor receptor-2. Absolute number of biopsies assigned to each clinicopathological subtype and CtIP/RBBP8 expression level. The number in brackets represents the percentage of samples within a given category of CtIP/RBBP8 expression.

Surprisingly, we found 40% of the samples had no detectable CtIP/RBBP8 expression. CtIP/RBBP8 is an essential gene in mammals [13] unless RB1 expression is also diminished [13]. Thus, we reasoned that the tumor samples without CtIP/RBBP8 expression might correlate with an altered expression of RB1. Lack of RB1 in many tumors has been widely proven [3], and breast cancer is not an exception [36–38]. Thus, we decided to analyze the presence of RB1 in the same breast samples we tested for CtIP/RBBP8 levels, to see if there was a correlation between their expression (Fig. 1 and Table 3). We detected three distinct cell types with respect to RB1 expression: samples with high nuclear RB1 levels, similar to noncancer samples from breast reduction surgery (Fig. S2); samples with no expression; and samples with a reduced RB1 expression. Analyzing all the cancer biopsies for RB1 and CtIP/RBBP8 patterns revealed a strong statistically significant correlation between RB1 and CtIP/RBBP8 (Table 3): in more than 90% of those samples in which CtIP/RBBP8 was absent, RB1 was also either absent or reduced. However, more than half of the samples with normal CtIP/RBBP8 expression also retained high RB1 expression levels. An intermediate situation was observed on the samples that had been determined to have low CtIP/RBBP8 levels.

**Table 3:** Correlation between CtIP/RBBP8 and RB1 expression in breast cancer biopsies

	Absence	Low	Normal	*P-*value
Retinoblastoma				
Normal	9 (6.4)	27 (23.9)	41 (54.7)	<0.0001
Intermediate	88 (62.9)	72 (63.7)	32 (42.7)	
Negative	43 (30.7)	14 (12.4)	2 (2.7)	

Absolute number of biopsies assigned to each RB1 level and CtIP/RBBP8 expression level. The number in brackets represents the percentage of samples within a given category of CtIP/RBBP8 expression.

**Figure 2:**
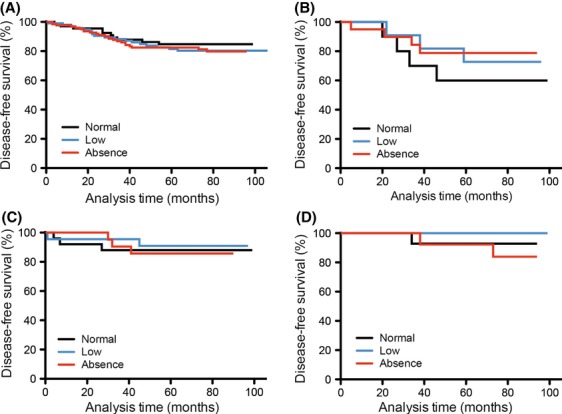
Disease-free survival and response to hormone therapy of patients in the cohort with respect to the CtIP/RBBP8 levels. (A) Overall representation of tumor relapse in all the patients of the cohort. Disease-free survival data were obtained from clinical records and plotted using the Kaplan–Meier method. The cohort was divided according to CtIP/RBBP8 levels as follows: level 2, normal expression (black line, *n* = 66); level 1, low expression (blue line, *n* = 102); level 0, no expression (red line, *n* = 128). (B) Kaplan–Meier representation of the time patients with luminal tumors treated with tamoxifen remained disease free. CtIP/RBBP8 levels: level 2, normal expression (black line, *n* = 10); level 1, low expression (blue line, *n* = 13); level 0, no expression (red line, *n* = 20). (C) Disease-free survival times in patients with luminal tumors treated with aromatase inhibitors. CtIP/RBBP8 levels: level 2, normal expression (black line, *n* = 25); level 1, low expression (blue line, *n* = 22); level 0, no expression (red line, *n* = 23). (D) Tumor relapse in patients with luminal tumors treated with a combination of tamoxifen and aromatase inhibitors. CtIP/RBBP8 levels: level 2, normal expression (black line, *n* = 14); level 1, low expression (blue line, *n* = 18); level 0, no expression (red line, *n* = 13).

**Figure 3:**
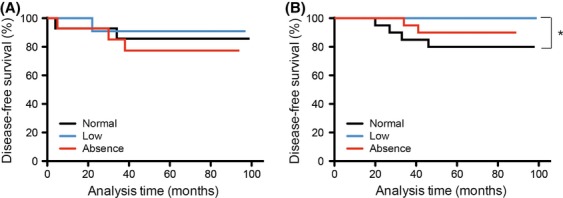
Relationship between the CtIP/RBBP8 levels and the response to chemotherapy for luminal cancers. (A) Disease-free survival times in patients with luminal tumors not treated with chemotherapy. CtIP/RBBP8 levels: level 2, normal expression (black line, *n* = 14); level 1, low expression (blue line, *n* = 12); level 0, no expression, (red line, *n* = 14). (B) Tumor relapse in patients with luminal tumors treated with chemotherapy as an adjuvant. An asterisk represents statistically representative changes (*P* < 0.05). CtIP/RBBP8 levels: level 2, normal expression (black line, *n* = 20); level 1, low expression (blue line, *n* = 18); level 0, no expression (red line, *n* = 21). In both cases (A and B), patients were treated with radiotherapy and hormone therapy.

As our data link CtIP/RBBP8 loss with more advanced and aggressive tumors, we next statistically analyzed the relationship between CtIP/RBBP8 expression levels and disease-free survival for patients for whom full clinical follow-ups were available (Figs. 2 and 3). First, we analyzed CtIP/RBBP8 levels at the time of biopsy and the interval of time during which the patients remained disease free; this revealed that there was no significant correlation between in tumor relapse and CtIP/RBBP8 protein levels (Fig. 2A). We then focused on luminal tumors, for which the CtIP/RBBP8 expression levels were evenly distributed between normal, low, and absence. It was previously reported that CtIP/RBBP8 silencing could be a mechanism of tamoxifen resistance [24]. We therefore analyzed the effects on CtIP/RBBP8 levels at the time of the biopsy in response to tamoxifen in luminal breast tumors. Patients in the studied cohort have been treated with either tamoxifen, aromatase inhibitors, or a combination of both. Analyzing these three groups independently, we observed no differences in disease-free survival, independent of the CtIP/RBBP8 levels in the three groups (Fig. 2B–D). All patients in the study with luminal tumors were treated with hormone therapy and radiotherapy. However, we could distinguish two groups depending on whether or not chemotherapy was used as an adjuvant. Thus, when we analyzed the disease-free interval on those categories, we observed that patients without a chemotherapeutical treatment responded in a similar way irrespectively of the CtIP/RBBP8 levels (Fig. 3A). However, a statistically significant correlation was observed for patients who were treated with chemotherapy as an adjuvant (Fig. 3B). Strikingly, low expression of CtIP/RBBP8 correlated with a lower proportion of tumor relapse as compared to patients with normal CtIP/RBBP8 levels (*P* < 0.05). A similar (but not statistically significant) trend was observed for samples with low CtIP/RBBP8 as compared to those in which the protein was absent. No differences were observed between tumors with normal CtIP/RBBP8 expression and those in which the protein was absent (nt).

## Discussion

CtIP/RBBP8 is a protein with a strong connection to cancer due to its functional and physical interactions with bona fide tumor suppressors. Although some studies have found CtIP/RBBP8 to be mutated in tumor samples [18–21], and some general screenings using RT-polymerase chain reaction (PCR) have found changes in its mRNA levels (Oncomine), we describe here the first systematic study of the presence of CtIP/RBBP8 at the protein level. We performed a retrospective study to correlate CtIP/RBBP8 expression with clinicopathological variables and disease-free survival. We found that the CtIP/RBBP8 level is indeed relevant in the appearance and prognosis of breast cancer. Thus, we propose that CtIP/RBBP8 itself should be considered as a tumor suppressor.

In agreement with our results, genome-wide screenings that analyzed CtIP/RBBP8 expression in different tumors at the level of mRNA using microarrays [24, 39–43] have also reported a correlation between high CtIP/RBBP8 expression and a positive ER mark. However, even though CtIP/RBBP8 mRNA was found to be overrepresented in certain tumors [12, 35], we have never observed overexpression at the protein level in breast cancer.

CtIP/RBBP8 overexpression was first identified in tumors linked with an increase of cyclin D1 transcription [35]. A different report also described CtIP/RBBP8 increases, which were, however, not significant [12]. Although we cannot discard that CtIP/RBBP8 protein levels are indeed increased in some samples, our data suggest that its contribution to cancer development is mainly due to its loss. CtIP/RBBP8 protein and mRNA expression are tightly controlled at many different levels, including gene transcription and protein degradation during the cell cycle [13, 35, 44–46]. Moreover, several protein modifications are involved in protein stability. Thus, mRNA expression does not always correlate with protein expression, and extrapolating CtIP/RBBP8 levels from mRNA data could be misleading. In fact, it is possible that this previously reported mRNA overexpression represents an attempt to increase protein levels that are abnormally low due to increased protein degradation.

Wu et al. [24] found that low levels of CtIP/RBBP8 at the time of diagnosis protect cancer cells from tamoxifen treatment. This observation prompted them to propose CtIP/RBBP8 silencing as a novel mechanism for tamoxifen resistance in breast cancer. They analyzed the response to tamoxifen of 59 nonoperable ER+ breast tumors and found that the lower the expression of CtIP/RBBP8, the lower the tumor size reduction. However, they did not study tumor relapse in the long term. In the cohort studied here, no differences to the response of hormone treatment were observed, for example, not from tamoxifen, aromatase inhibitors, or a combination of both, in terms of long-term disease-free survival. Therefore, we propose that breast cancers with low levels of CtIP/RBBP8 might respond worse to tamoxifen treatment, but that once those tumors were resected, the probability of tumor relapse for those patients treated with tamoxifen was the same, irrespective of the level of CtIP/RBBP8 expression detected in the biopsy.

Strikingly, when we analyzed the response of luminal cancers to a coadjuvant treatment with chemotherapy, we discovered that patients with low levels of CtIP/RBBP8 responded better (Fig. 3B). All 18 of those patients remain disease free for as long as the study took place. Therefore, although lower CtIP/RBBP8 expression correlated with more aggressive tumors at the time of diagnosis, treatment is more effective in this specific subset. Most chemotherapeutic agents used as coadjuvant base their action in artificially creating DNA damage, either directly or by increasing replication stress [47]. CtIP/RBBP8 is an important player in the response to DNA damage; indeed, CtIP/RBBP8 depletion renders cells sensitive to those agents, such as camptothecin or VP16 [16]. Thus, we conclude that cells with reduced overall levels of CtIP/RBBP8 are responding better to the chemotherapy due to this increased sensitivity. In fact, we propose that studying CtIP/RBBP8 expression could be used as a marker to define which cancers should be treated with chemotherapeutic agents. Surprisingly, this sensitivity is partially lost in cells that do not express any CtIP/RBBP8, and patients with no CtIP/RBBP8 expression behave intermediately between those classified in low and normal CtIP/RBBP8 levels. There can be several alternative explanations for this finding. First, it is possible that the total absence of CtIP/RBBP8 activates alternative DNA repair pathways that can (at least partially) handle the DNA damage caused by these therapeutical agents. Second, although RB1 is reduced in those cancers without CtIP/RBBP8, it is likely that the cell cycle progression is affected. As many of the chemotherapeuticals act during S-phase, it would not be surprising that the amount of DNA lesions created is greatly reduced for cancers without CtIP/RBBP8, rendering the drugs less effective. Finally, the intermediate trend observed for tumors in which CtIP/RBBP8 is absent could reflect the small numbers of biopsies analyzed (*n* = 21). It is possible that a larger study would find that patients with no CtIP/RBBP8 expression are also more sensitive to chemotherapy.

In conclusion, we have performed a retrospective study of the relationships between CtIP/RBBP8 expression levels and cancer prognosis and relapse using paraffin-embedded breast cancer biopsies from a cohort of 384 patients. Our results suggest a strong relationship between no or low expression of CtIP/RBBP8 and poor breast cancer prognosis. On the other hand, CtIP/RBBP8 is a poor marker for predicting the overall response to treatment and cancer and disease-free survival. However, low levels of CtIP/RBBP8 increase the response to chemotherapy in luminal cancers when combined with hormone therapy and radiotherapy.

## Supporting Information

**Figure S1.** CtIP/RBBP8 antibody specificity for immunostaining. U2OS cells were transfected with a previously characterized siRNA against CtIP/RBBP8 (siCtIP) or a control siRNA (siCtrl) [16] and immunostained with anti-CtIP antibody.

**Figure S2.** CtIP/RBBP8 expression in normal mammary gland. (A) Paraffin-embedded normal breast tissue was immunostained with CtIP/RBBP8 antibody and hematoxylin as described in the Patients and Methods section. (B) A representative image of each CtIP and RB expression category is shown for comparison. From left to right categories 2, 1, and 0 (normal, low, or no expression of CtIP/RBBP8 and RB1). Compare nuclear staining with (A).
