# Supplementary figures and images for: Prognostic value of CtIP/RBBP8 expression in breast cancer
Source: Cancer Med. 2013 Oct 3;2(6):774–83. doi: 10.1002/cam4.141 (PMC3892382; doi:10.1002/cam4.141)

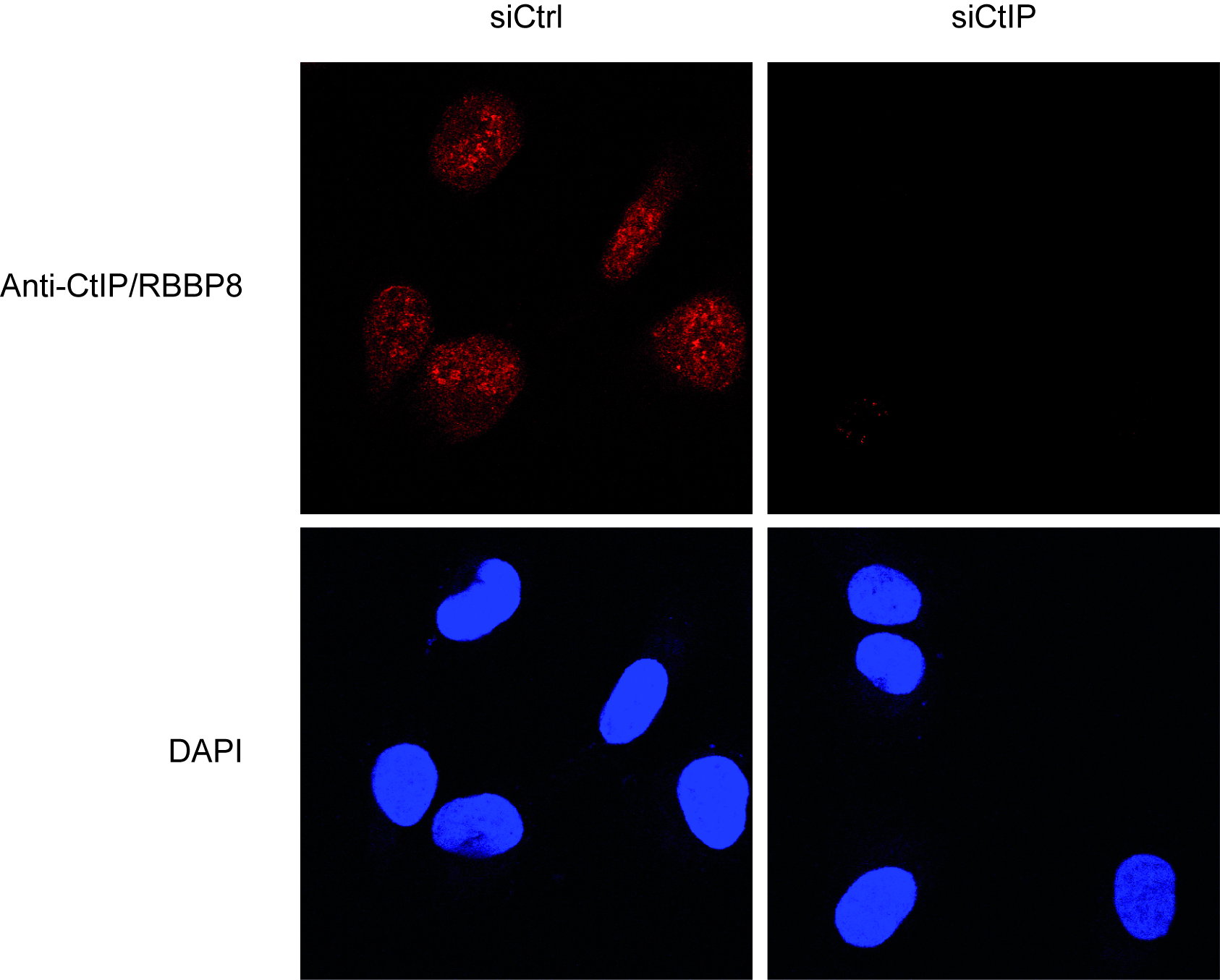

Supplement: Supplementary file 1 [file cam40002-0774-SD1.tif]

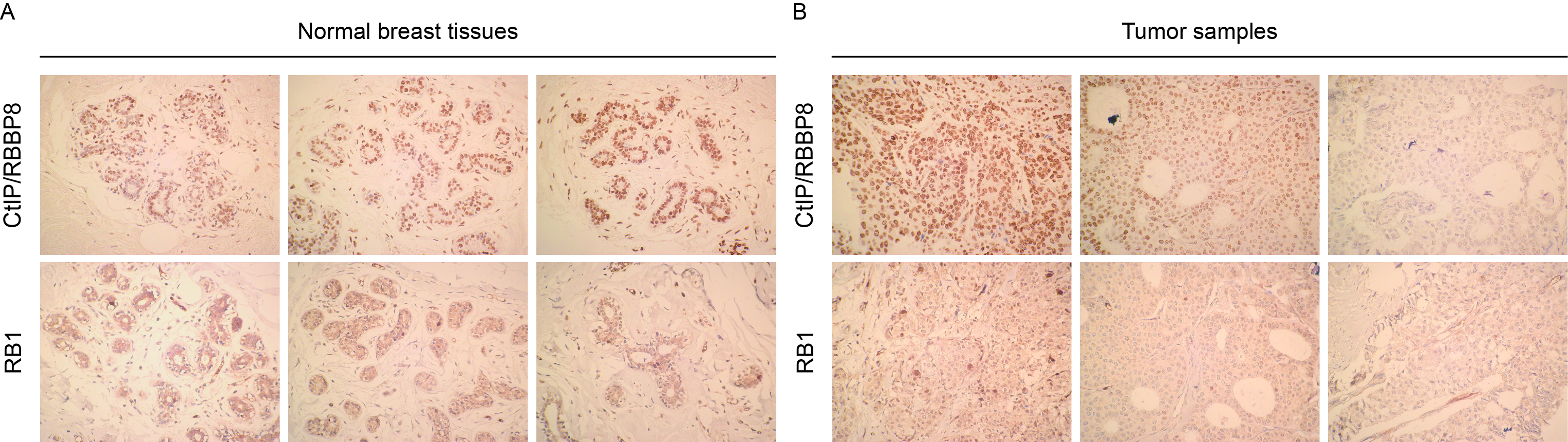

Supplement: Supplementary file 2 [file cam40002-0774-SD2.tif]
